# Proteomics Analysis Reveals Non-Controlled Activation of Photosynthesis and Protein Synthesis in a Rice *npp1* Mutant under High Temperature and Elevated CO_2_ Conditions

**DOI:** 10.3390/ijms19092655

**Published:** 2018-09-07

**Authors:** Takuya Inomata, Marouane Baslam, Takahiro Masui, Tsutomu Koshu, Takeshi Takamatsu, Kentaro Kaneko, Javier Pozueta-Romero, Toshiaki Mitsui

**Affiliations:** 1 Graduate School of Science and Technology, Niigata University, 2-8050 Ikarashi, Niigata 950-2181, Japan; f14m005a@mail.cc.niigata-u.ac.jp (T.I.); zoujoukikou@gmail.com (T.M.); tsutomu58koshu@hotmail.co.jp (T.K.); ttakamatsu@agr.niigata-u.ac.jp (T.T.); k-neko@gs.niigata-u.ac.jp (K.K.); 2 Department of Biochemistry, Niigata University, Niigata 950-218, Japan; mbaslam@gs.niigata-u.ac.jp; 3 Instituto de Agrobiotecnología (CSIC, UPNA, Gobierno de Navarra), Mutiloako Etorbidea Zenbaki Gabe, 31192 Mutiloabeti, Nafarroa, Spain; javier.pozueta@unavarra.es

**Keywords:** chloroplast, elevated CO_2_, heat stress, nucleotide pyrophosphatase/phosphodiesterase, (phospho)-proteomics, photosynthesis, protein phosphorylation, 14-3-3 proteins, *Oryza sativa* L., starch, sucrose

## Abstract

Rice nucleotide pyrophosphatase/phosphodiesterase 1 (NPP1) catalyzes the hydrolytic breakdown of the pyrophosphate and phosphodiester bonds of a number of nucleotides including ADP-glucose and ATP. Under high temperature and elevated CO_2_ conditions (HT + ECO_2_), the *npp1* knockout rice mutant displayed rapid growth and high starch content phenotypes, indicating that *NPP1* exerts a negative effect on starch accumulation and growth. To gain further insight into the mechanisms involved in the *NPP1* downregulation induced starch overaccumulation, in this study we conducted photosynthesis, leaf proteomic, and chloroplast phosphoproteomic analyses of wild-type (WT) and *npp1* plants cultured under HT + ECO_2_. Photosynthesis in *npp1* leaves was significantly higher than in WT. Additionally, *npp1* leaves accumulated higher levels of sucrose than WT. The proteomic analyses revealed upregulation of proteins related to carbohydrate metabolism and the protein synthesis system in *npp1* plants. Further, our data indicate the induction of 14-3-3 proteins in *npp1* plants. Our finding demonstrates a higher level of protein phosphorylation in *npp1* chloroplasts, which may play an important role in carbohydrate accumulation. Together, these results offer novel targets and provide additional insights into carbohydrate metabolism regulation under ambient and adverse conditions.

## 1. Introduction

Changes in climate explain a major portion (32–39%) of yield variability, which correspondingly may translate into large fluctuations in global crop production [1]. Climate change that alters photosynthetic rates may modify physiological responses, plant growth rates (and overall productivity), and resource uses. Therefore, the potential for physiological functioning to evolve in response to climate change will be a key indicator of plant resilience in future environments [2,3]. As they are mostly immobile, plants must adapt to their biotic and abiotic environments limiting or promoting productivity and carbon biosequestration. Photosynthesis can be critical to mitigating the effects of changing climatic conditions. Although the relationship between stomatal conductance, CO_2_ uptake, and photosynthesis fluctuates in nature, leaf photosynthesis is known to be highly correlated with stomatal conductance in rice [4,5]. Studies employing mutants deficient in a stomatal anion channel protein SLAC1 (Slow Anion Channel-associated 1) revealed that stomatal conductance is a major determinant of photosynthetic rate in higher plants [6,7]. Thus, terrestrial plants regulate CO_2_ uptake by stomatal switch in response to environmental and biochemical stimuli. The assimilated carbon is further converted to starch in the plastid stroma or to sucrose in the cytosol via sugar nucleotides. In rice leaves, it has been shown that the assimilated carbon is partitioned into sucrose rather than starch [8,9]. Under varying environmental conditions, plant growth and development have to be counterbalanced for defense and stress adaption. Notably, proportional changes in protein composition in response to environmental changes are a major cellular response to requirements of homeostatic adjustment and metabolic remodeling.

Starch is synthesized in plant leaves during the day from photosynthetically fixed carbon. It is then mobilized and thus constitutes an important carbon reserve as well as an energy source for plants and provides the major nutritional value of food crops. Starch biosynthesis occurs in plastids and requires several key enzymes [10,11]. Starch, probably the most important metabolite of plant carbohydrate metabolism, is an insoluble polyglucan produced by starch synthase (SS) using ADP-glucose as the sugar donor nucleotide. Among the many enzymes participating in plant nucleotide catabolism, the pyrophosphatase/phosphodiesterase (NPP) family members, including *NPP1*, actively catalyze the hydrolytic breakdown of the pyrophosphate and phosphodiester bonds of ADP-glucose [12,13,14]. In rice, six *NPP* genes (*NPP1–6*) have been identified and characterized [14]. The *NPP* genes are located on chromosomes 3 (*NPP3*), 8 (*NPP1*), 9 (*NPP6*), and 12 (*NPP2*, *4*, *5*) in the rice genome. Although *NPP3* was exclusively localized in the endomembrane system [15], *NPP1*, 2, and 6 exhibited a dual localization in both the plastid and endomembrane systems [13,16,17], as has been found for other plastidial glycoproteins [18,19,20,21,22]. Whereas *NPP1* and *NPP6* recognized nucleotide sugars, *NPP2* did not recognize these compounds as substrates but preferentially hydrolyzed uridine diphosphate (UDP), ADP, and adenosine 5′-phosphosulfate (APS). *NPP1* best hydrolyzed ADP-glucose, ADP-ribose, and ATP, while ADP and ADP-glucose were the best substrates for *NPP6*. Rice *NPP* genes showed tissue- and stage-specific expression [14,15]. Thus, it is possible to suppose that rice NPPs are engaged in multiple biological functions through the influence of the turnover of nucleotides and nucleotide derivatives. *NPP1* is a major determinant of ADP-glucose pyrophosphatase (AGPPase) activity and can reduce the plastidic pool of ADP-glucose required for starch accumulation in rice [14]. Plant NPPs seem to play a crucial role in carbon flux by transporting carbon taken up from starch and from cell wall polysaccharide biosynthesis to other metabolic pathways in response to the physiological needs of the cell. We have previously shown that when the *npp1* null mutant is grown under different CO_2_ concentration and temperature conditions, *NPP1* exerts a negative effect on plant growth and starch accumulation. This provided the first in vivo evidence for the role of *NPP1* in the control of growth and reserve pool of carbohydrate in rice under fluctuating climatic conditions [14]. Recently, the presence of NPPs in the plastidial compartment was further confirmed [13,15,16]. Moreover, we have tentatively identified complex-type and pauci-mannosidic-type oligosaccharide chains with β1,2-xylose and/or differential core α1,3-fucose residue(s) in rice *NPP1*, which provides strong evidence that the trans-Golgi compartments participate in the Golgi-to-plastid trafficking and targeting mechanism of *NPP* in rice [16]. Plants with a defective endoplasmic reticulum (ER) *N*-glycosylation pathway and *N*-glycan maturation are associated with diverse phenotypes and are more sensitive to environmental conditions [23]. Nonetheless, the regulatory mechanisms of high temperature and elevated CO_2_-induced starch accumulation and growth stimulation in *npp1* leaves are unknown. Herein, we highlight that the loss-of-function of *NPP1* not only influences stomatal conductance, photosynthesis, and plants’ storage of starch and sucrose but also impacts a set of proteins involved in carbohydrate metabolism and the protein synthetic system in rice leaves under high temperature and CO_2_ conditions (HT + ECO_2_). 

## 2. Results and Discussion

### 2.1. Knocking out NPP1 Decreases Leaf Temperature and Enhances Stomatal Conductance under High Temperature and Elevated CO_2_ Conditions

**Figure 1:**
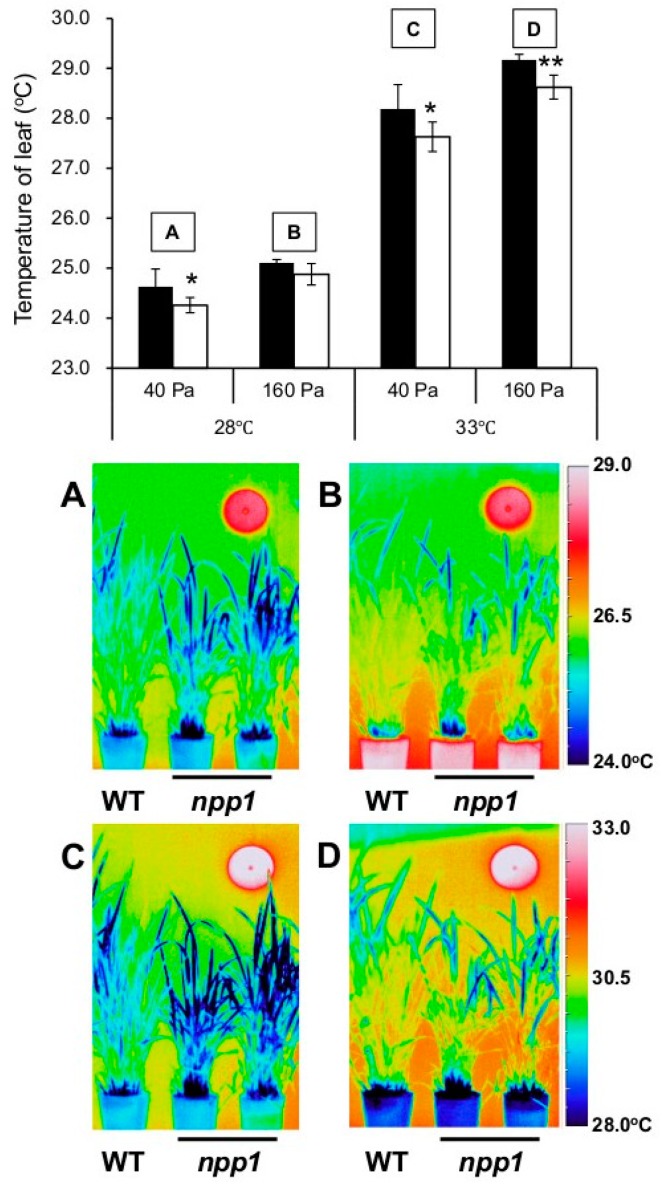
Leaf temperatures of WT and *npp1* mutant plants under different temperatures and CO_2_ concentrations in Biotron’s environmental conditions. WT (filled bars) and *npp1* (open bars) plants grown for 60 days under normal conditions (14 h light/10 h dark: 28/23 °C, 40 Pa CO_2_) after germination were subjected to thermographic analysis of their leaf temperatures under different temperature and CO_2_ conditions: (**A**) 28 °C, 40 Pa, (**B**) 28 °C, 160 Pa, (**C**) 33 °C, 40 Pa, and (**D**) 33 °C, 160 Pa. Values in upper panel show the means ± standard deviation (s.d.) (*n* = 5). Asterisks indicate significant differences by Student’s *t*-test (*, *p* > 0.05; **, *p* > 0.01). Lower panels represent the thermographic images. WT: wild-type.

**Figure 2:**
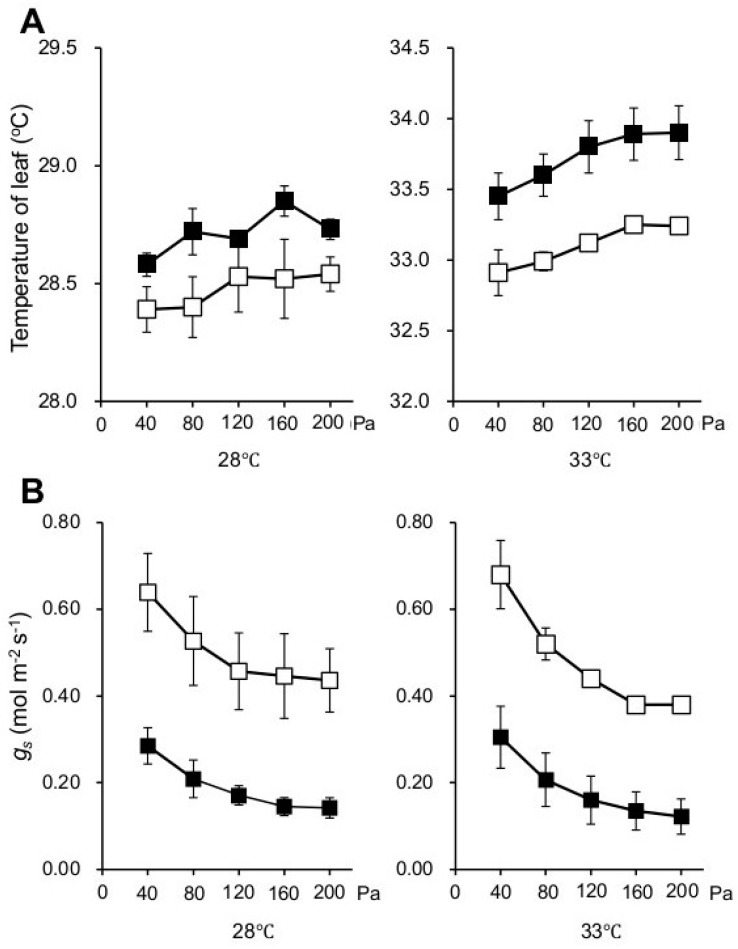

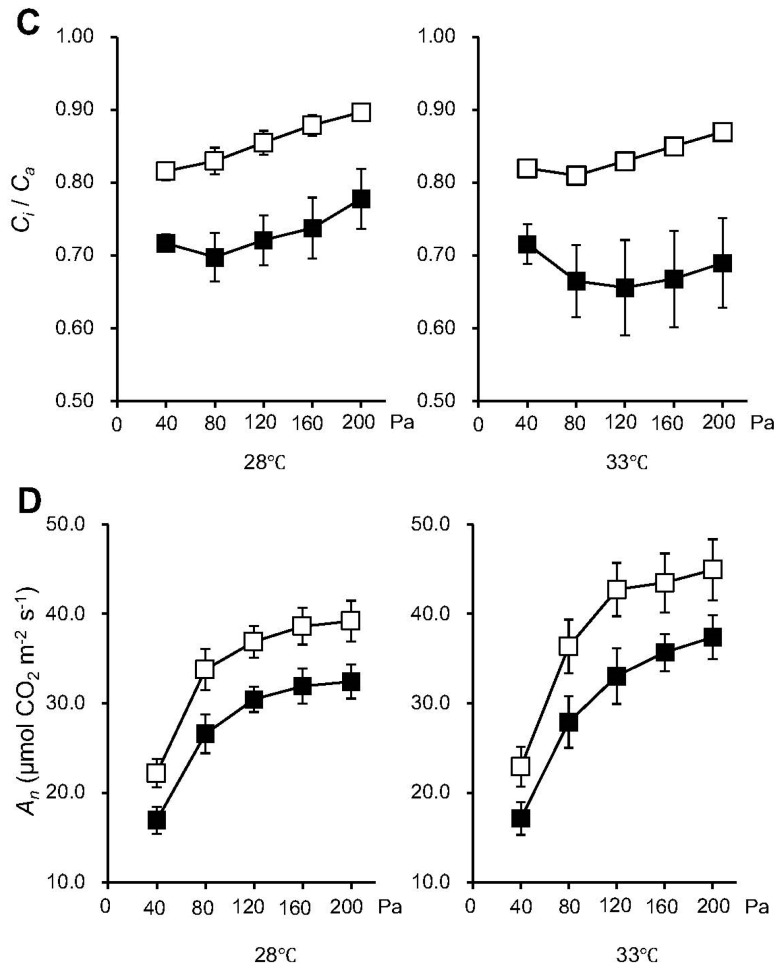
Changes in temperatures, *g_s_*, *C_i_*/*C_a_*, and *A_n_* of WT and *npp1* mutant plants under different temperatures and CO_2_ concentrations in a controlled growth chamber. Top leaves of main culm of WT (■) and *npp1* (□) plants grown for 60 days under normal conditions (14 h light/10 h dark: 28/23 °C, 40 Pa CO_2_) were subjected to measurements of temperature (**A**), stomatal conductance to water (*g_s_*) (**B**), ratios of internal [CO_2_] to ambient [CO_2_] (*C_i_*/*C_a_*) (**C**), and photosynthetic rates (*A_n_*) (**D**) under the following conditions: temperature, 28 and 33 °C; CO_2_, 40 to 200 Pa; relative humidity, 50%; light, 1000 μmol·photons·m^−2^·s^−1^. Values show the means ± s.d. (*n* = 3).

In order to examine phenotypic characteristics of the *npp1* mutant, 60-day old wild-type (WT) and *npp1* mutant rice plants were subjected to thermographic analysis in a spacious Biotron (length 4.5 × width 4.5 × height 1.8–2.2 m) with varying temperatures (28 and 33 °C) and CO_2_ concentrations (40 and 160 Pa) under natural sunlight. As shown in Figure 1, the leaf temperatures of WT and *npp1* plants increased concomitantly with the CO_2_ concentration irrespective of temperature conditions. Notably, the leaf temperature in *npp1* plants was apparently lower than in WT plants at all temperature and CO_2_ conditions examined (Figure 1). We further examined the flag leaves’ temperatures in WT and *npp1* under the different temperature (28 and 33 °C) and CO_2_ conditions (40 to 200 Pa) under fully controlled environmental chamber conditions (width 64 × width 56 × height 108 cm) using a portable photosynthesis measuring system (Figure 2A). The leaf temperatures of both WT and *npp1* gradually increased as CO_2_ concentrations increased, irrespective of temperature conditions, and reached saturation at 160 Pa (Figure 2A).

The reduction of leaf temperature in *npp1* strongly suggested that knocking out *NPP1* increases the transpiration rate of the plant. This inference was corroborated by the analysis of the stomatal conductance (*g_s_*) under varying temperatures and CO_2_ concentrations. As shown in Figure 2B, elevated CO_2_ concentrations caused partial stomatal closure in the two genotypes. No significant differences were observed in the *g_s_* values between the two temperature regimes. Notably, the *g_s_* values in *npp1* plants were higher than in WT plants under all temperatures and CO_2_ concentrations. 

Previous reports have shown that high CO_2_ decreases *g_s_* in a temperature-independent manner [6,24,25,26,27,28]. Changes in *g_s_* value in the *npp1* mutant under different temperature and CO_2_ conditions were similar to those in WT, indicating that the stomatal regulatory mechanisms of *npp1* work normally.

### 2.2. Knocking out NPP1 Enhances Photosynthetic Capacity under High Temperature and Elevated CO_2_ Conditions

The high *g_s_* values observed in the *npp1* mutant suggested that the lack of *NPP1* expression enhances CO_2_ diffusion. In line with this presumption we found that, irrespective of the temperature regime, the intercellular-to-atmospheric CO_2_ mole fraction (*C_i_*/*C_a_*) in *npp1* was higher than in WT plants at all atmospheric CO_2_ concentrations (Figure 2C). Consistently, *npp1* plants had higher net rates of CO_2_ assimilation (*A_n_*) than WT plants at all atmospheric CO_2_ levels in the two temperature regimes (Figure 2D). 

**Figure 3:**
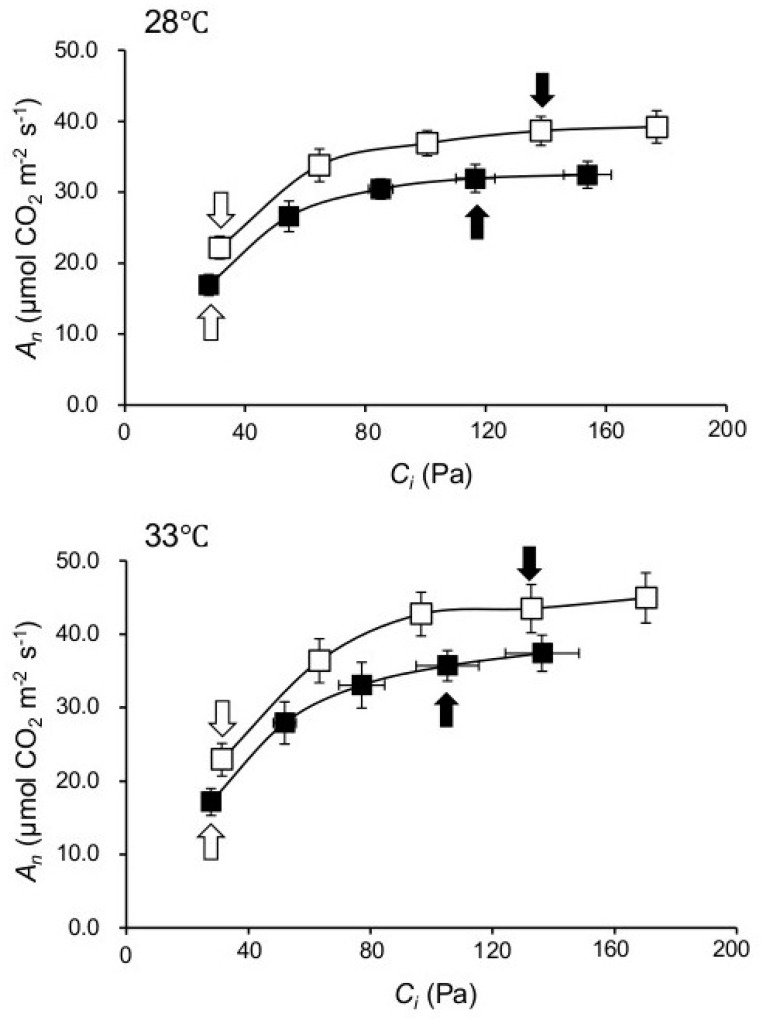
Photosynthetic rates at different intracellular [CO_2_] (*A_n_* /*C_i_* curve) of leaves from WT (■) and *npp1* (□) plants. The results of Figure 2 were used to draw an *A_n_*/*C_i_* curve. Open and filled arrows represent the data obtained at *C_a_* of 40 and 160 Pa, respectively.

**Figure 4:**
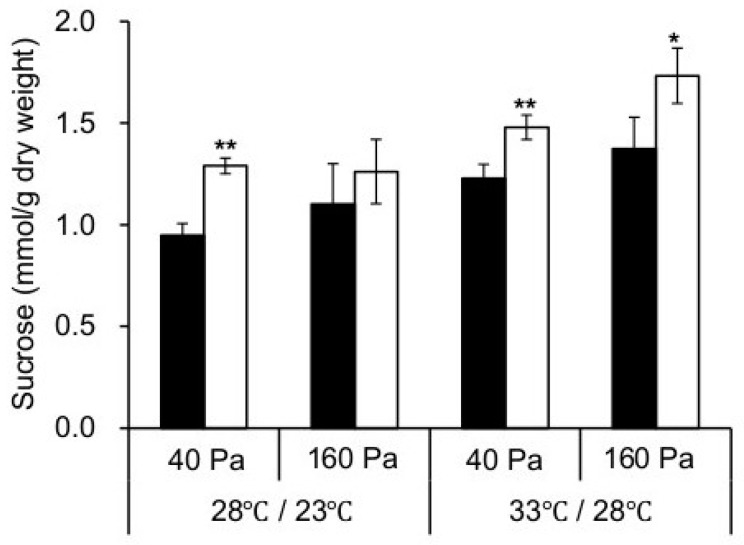
Sucrose accumulation in leaves of WT and *npp1* mutant plants. WT (filled bars) and *npp1* (open bars) plants grown for 60 days under normal conditions (28/23 °C, 40 Pa CO_2_) were further incubated under different temperatures and CO_2_ concentrations. At the end of a light cycle, the leaves of the WT and the *npp1* mutant plants were subjected to sucrose assays. Values show the means ± s.d. (*n* = 3~5). Asterisks indicate significant differences by Student’s *t*-test (*, *p* > 0.05; **, *p* > 0.01).

Whether enhanced photosynthesis in *npp1* plants is solely the consequence of enhanced CO_2_ diffusion was investigated by measuring *A_n_* under varying *C_i_*. As shown in Figure 3, these analyses revealed that the *npp1* mutant showed higher *A_n_* values than WT plants at all *C_i_* levels. In rice plants, sucrose is the primary transport sugar and plays a central role in plant growth and development. As expected, levels of sucrose in *npp1* mutants were found to be significantly higher compared with their control WT in any incubation conditions (Figure 4). Previously, the *npp1* mutants have been shown to accumulate starch under high CO_2_ concentration conditions [14]. Thus, it is evident that *NPP1* exerts a negative effect on carbohydrate accumulation under HT + ECO_2_. In these conditions, the *npp1* mutants increase photosynthesis and the Calvin-Benson cycle (as detailed below), the latter via increased accumulation of Rubisco activase, enabling *npp1* plants to adjust downstream reactions such as sucrose biosynthesis and phloem loading to the increased leaf assimilation. These results indicate that the stimulation of photosynthesis in the *npp1* mutant was caused by both stomatal conductance and non-stomatal conductance related causes. Stomata opening and closure occurs via changes in the turgor pressure of guard cells, and the physiological event is regulated by ion and sugar movements in the guard cells [6,29,30]. Increased CO_2_ concentrations enhance anion channel activity (which has been proposed to be a means of mediating the efflux of osmoregulatory anions from guard cells) [31], causing stomatal closure in rice leaves (Figure 2B, [32]). In *npp1* guard cells, the accumulation of sucrose probably causes stomata opening, further activating the photosynthesis of *npp1* leaves under the elevated CO_2_. The *NPP1* enzyme, which hydrolyzes sugar nucleotides and ATP, is localized and functions in the chloroplast of rice, therefore, the contents of ATP in chloroplasts should be increased. The high level of ATP in *npp1* chloroplasts would boost the turnover of the Calvin cycle and actively convert CO_2_ to starch and sucrose. In fact, in *npp1* null mutants grown under different temperature conditions and CO_2_ concentrations, a negative effect on the reserve pool of carbohydrates is seen. In this case, *NPP* activity may occupy a central position in carbon metabolism and its metabolic output to provide products such as the amino acids and lipids necessary for increasing plant biomass.

### 2.3. Proteomic Characterization of npp1 Leaves under High Temperature and Elevated CO_2_ Conditions

To characterize changes in the proteome of *npp1* leaves under HT + ECO_2_, we carried out a quantitative proteomic analysis. Proteins extracted from leaves of WT and *npp1* plants grown under normal (28/23 °C and 40 Pa CO_2_) and HT + ECO_2_ (33/28 °C and 160 Pa CO_2_) conditions were labeled by iTRAQ (isobaric tag for relative and absolute quantitation), followed by tandem mass spectrometry (MS/MS) analysis. Using this approach, 103 differentially expressed proteins were successfully identified among 1701 detected proteins in total. The general trend indicates that the response of the *npp1* mutant to the HT + ECO_2_ treatment is due, at least partly, to changes in the expression of proteins from the following groups: photosynthesis, carbohydrate metabolism, protein synthesis, and signaling.

### 2.4. Photosynthesis and Carbohydrate Metabolism

**Figure 5:**
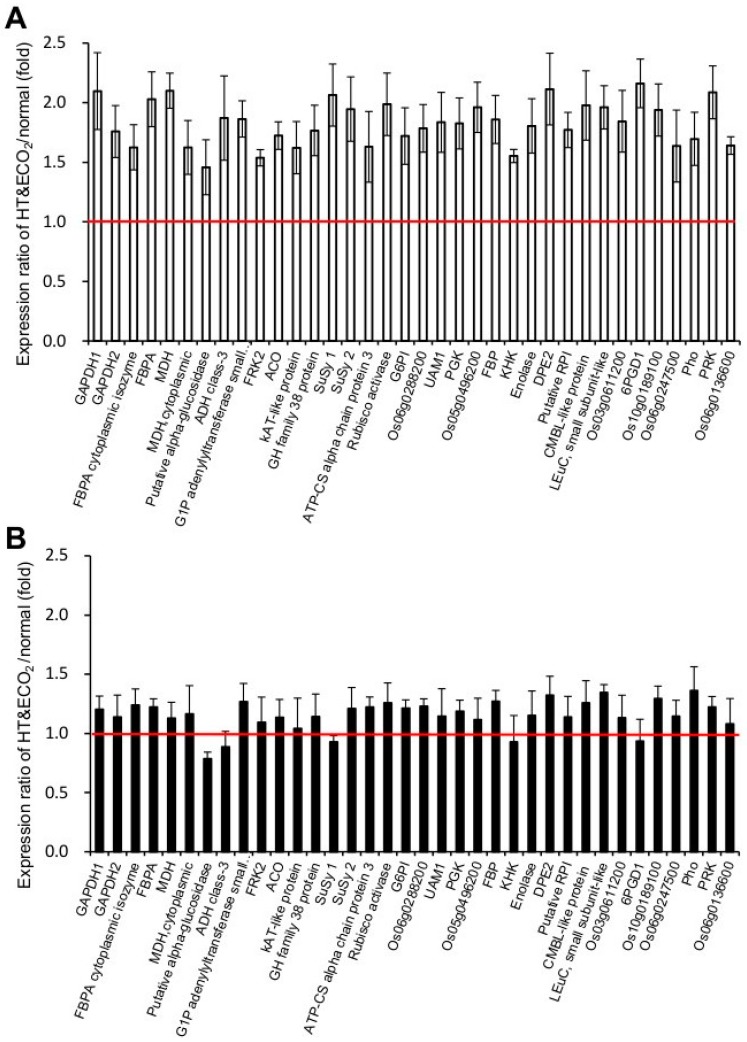

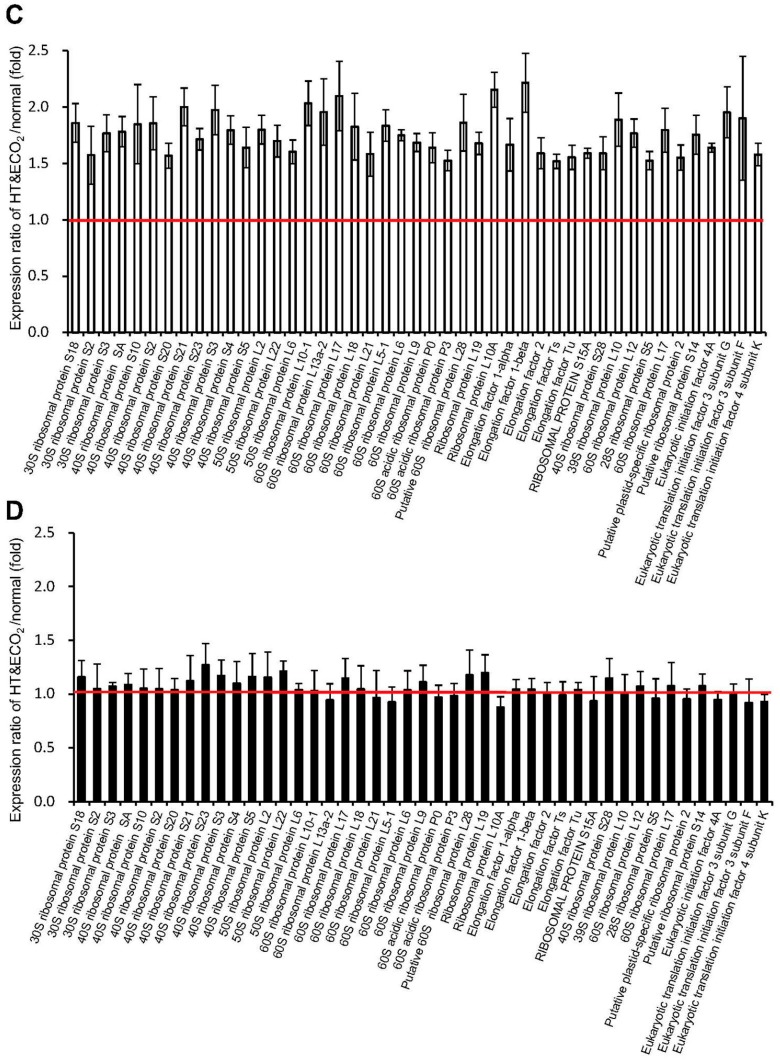
Changes in the expression of carbohydrate- and protein synthesis-related proteins in leaves of WT and *npp1* mutant plants under high temperature and elevated CO_2_ concentrations. The leaves of WT (**B**,**D**) and *npp1* (**A**,**C**) plants were incubated under normal (28/23 °C, 40 Pa CO_2_) and HT + ECO_2_ (33/28 °C, 160 Pa CO_2_) conditions, and then subjected to a proteomic analysis with Isobaric tags for relative and absolute quantitation (iTRAQ) labeling. Values show the means ± s.d. (*n* = 3). The red line shows the ratio between HT+ECO_2_/normal condition mean equal to 1.

Various proteins associated with photosynthesis and carbohydrate metabolism were upregulated in HT + ECO_2_ grown *npp1* plants (Figure 5A) compared with WT plants (Figure 5B). The HT + ECO_2_ treatment promoted the expression of Rubisco activase, a protein that acts as Rubisco’s catalytic chaperone [33]. This result is consistent with the enhanced photosynthetic carbon assimilation and also a rise in electron transport capacity. Growth under HT + ECO_2_ also upregulated the expression of Calvin-Benson enzymes (e.g., fructose-bisphosphate aldolase (FBPA), phosphoglycerate kinase (PGK), and phosphoribulokinase (PRK)). The data presented thus indicate that the HT + ECO_2_ enhancement of photosynthesis in *npp1* is the result of enhanced enzymatic activities involved in CO_2_ fixation.

The starch synthesis-related enzymes, G1P adenylyltransferase (AGPase), sucrose synthase (SuSy), and 4-α-glucanotransferase protein (DPE2), exhibited a clear upregulation under HT + ECO_2_. AGPase is produced from ATP and glucose 1-phosphate to the ADP-glucose necessary for starch biosynthesis. SuSy is responsible for the conversion of sucrose and a nucleoside diphosphate into the corresponding nucleoside diphosphate glucose and fructose, however, this sucrolytic protein may participate in the direct conversion of sucrose into ADP-glucose linked to starch biosynthesis. The sucrose and starch metabolic pathways are tightly interconnected by means of cytosolic ADP-glucose producing enzymes such as SuSy and by the action of an ADP-glucose translocator located on the chloroplast envelope membranes [10,34]. Furthermore, the levels of cytosolic glucanotransferase DPE2 involved in the conversion of starch into sucrose [35] were high in *npp1* leaves under HT + ECO_2_ conditions. The high expression of AGPase, SuSy, and DPE2 induced under HT + ECO_2_ conditions would be crucial factors for carbohydrate accumulation. 

### 2.5. Protein Synthesis System

The marked upregulation of a large set of proteins related to the protein synthesis system when knocking out *npp1* was also produced by the enhancement of temperature and CO_2_ (Figure 5C), although such an increase was not observed in WT leaves (Figure 5D). Ribosome components such as 60S, 50S, 40S, and 30S ribosomal proteins were identified as being upregulated. In addition, elongation factor 1 (EF-Iα and β), 2, T (EF-Ts and -Tu), and four rice eukaryotic initiation factor proteins (eIF 3A, eIF 3F, eIF4A, and eIF4F) were increased in *npp1* under HT + ECO_2_. EF-Iα controls GTP-binding proteins responsible for cytoskeletal tubulin, which is positively correlated with starch accumulation [36]. Early studies showed that EF-Iα can influence the organization of cytoskeletal components surrounding protein bodies around the endoplasmic reticulum membranes, which appear to reorganize coincident with the actively accumulating storage proteins in the endosperm [37]. eIF participates in most translation initiation processes and plays important roles in the growth and development (and organ size) of *Arabidopsis* and rice [38,39,40].

### 2.6. Signaling

**Figure 6:**
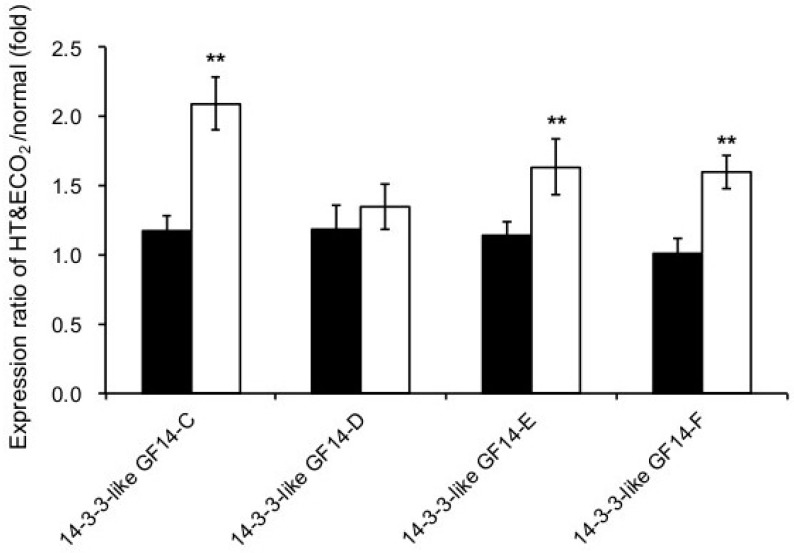
Changes in expression of 14-3-3 proteins in leaves of WT and *npp1* mutant plants under high temperature and elevated CO_2_ concentrations. Details of incubation conditions via a proteomic analysis were described in Figure 5. The leaves of WT (filled bars) and *npp1* (open bars) plants were incubated under normal and HT + ECO_2_ conditions. Values show the means ± s.d. (*n* = 3). Asterisks indicate significant differences by Student’s *t*-test (**, *p* > 0.01).

Several 14-3-3 proteins exhibited significant changes in *npp1* mutants under HT + ECO_2_ conditions (Figure 6) and were thus deemed to play important roles in starch biosynthesis in rice leaves. In this study, the upregulation of three 14-3-3 isoforms (14-3-3 GF14C, 14-3-3 GF14E, and 14-3-3 GF14F) was detected, suggesting changes in the phosphorylation status in *npp1* mutants. Recently, the ectopic overexpression of the cassava 14-3-3 gene in *Arabidopsis* showed an increase in starch content in the leaves from transgenic plants [36]. A striking feature of the 14-3-3 proteins is their ability to bind a multitude of functionally diverse signaling proteins, including kinases, phosphatases, and transmembrane receptors [41], and 14-3-3 proteins play roles in regulating plant development and stress responses by effecting direct protein-protein interactions [42,43,44]. The interactions with 14-3-3s are subject to environmental control through signaling pathways that impact on 14-3-3 binding sites. The phosphoserine/threonine-binding 14-3-3 proteins participate in environmentally responsive phosphorylation-linked regulatory functions in plants and are potentially involved in starch regulation [45]. Previous studies have identified the protein-protein interactions between 14-3-3 and key enzymes of primary metabolism (e.g., sucrose-phosphate synthase and glyceraldehyde-3-phosphate dehydrogenase) and showed the central role of 14-3-3s as regulators of enzymes of cytosolic metabolism and ion pumps [46,47,48,49]. A molecular genetic analysis of 14-3-3 isoforms using overexpressed and knockout plants with studies of protein-protein interactions revealed alterations in the level of metabolic intermediates of glycolysis, tricarboxylic acid (TCA), and biosynthesis of aromatic compounds [50]. Hence, we may speculate that 14-3-3 may play a role in the accumulation of starch in *npp1* mutants by interacting with metabolic enzymes and thus appears to maintain those enzymes in an active state in the cell. Notably, 14-3-3 proteins have been localized to the chloroplast stroma and the stromal side of thylakoid membranes [51], thereby implicating a potential role in starch regulation.

Photosynthesis, sugar assays, and quantitative proteomic analyses of leaves of knockout *npp1* revealed that the mutant plants always become highly active regardless of normal or HT and ECO_2_ conditions (Figure 3, Figure 4 and Figure 5). The higher *A_n_* in *npp1* mutants could be the consequence of various factors: (i) higher CO_2_ diffusion; (ii) more efficient conversion of light energy into ATP; and (iii) the upregulation of photosynthetic enzymes (Rubisco activase and Calvin-Benson enzymes). We consider that an activation effector would be the high level of ATP in chloroplasts, since *NPP1* preferentially hydrolyzes ATP [14]. Of note, a series of 14-3-3 proteins were upregulated in *npp1* mutants (Figure 6), suggesting that the protein phosphorylation status is possibly changed by the disruption of the *NPP1* gene. Phosphorylation of proteins in chloroplasts plays a major role in regulating both the light and dark reactions of photosynthesis. Light-harvesting chlorophyll a/b binding proteins [52,53,54], Rubisco [55], Rubisco activase [56], phosphoglycerate kinase [57], Glyceraldehyde 3-phosphate dehydrogenase (GAPDH) [58], and transketolase [59] were shown to be phosphorylated and the enzyme functions were regulated. In addition, sigma factor [60] and 24/28 RNA binding proteins [61] were also phosphorylated. 

We analyzed the phosphorylation state of WT and *npp1* chloroplast proteins under HT and ECO_2_ conditions by employing TiO_2_ chromatography and mass spectrometry. The phosphopeptides derived from chlorophyll a/b binding protein, Rubisco large chain, pyruvate phosphate dikinase, and some other plastidial enzymes and proteins were detected with good reproducibility. The level of protein phosphorylation in *npp1* chloroplasts under HT and ECO_2_ was higher than that of *npp1* under the normal condition or the WT chloroplasts (Table 1). The chlorophyll a/b binding protein is the main component of the light-harvesting complex (LHC), which is a light receptor that captures and delivers excitation energy to photosystems PSI and PSII. The reversible phosphorylation of light harvesting chlorophyll a/b binding proteins (LHCII) has been observed, which regulates state transitions for balancing the excitation energy between PSI and PSII [54,62]. As shown in Table 1, several phosphorylation sites of chlorophyll a/b binding proteins in the *npp1* mutant were more highly phosphorylated in comparison with the WT chloroplasts. In addition, the phosphorylation level of chlorophyll a/b binding proteins in both *npp1* and WT were increased under HT and ECO_2_ conditions. In our phosphoproteomic analysis, two phosphopeptides plastid movement impaired1 (PMI1) and plastid transcriptionally active 16 (pTac16) were found to be more abundant under ECO_2_ + HT condition in *npp1* mutant plants. The PMI1 is a plant-specific C2-domain protein that plays a role in organelle movement and positioning [63]. The movements of *npp1* organelles (i.e chloroplasts) within the cell, which become appropriately positioned under ECO_2_ + HT, could be a fundamental cellular activity to accomplish their functions and adapt to environmental stress. The pTac16 is a plastid membrane-attached multimeric protein complex involved in plastid transcription and translation. The knockout lines *ptac* seedlings developed white or yellow cotyledons, failed to accumulate chlorophyll even under low light intensities, impaired plastid structure [64], and downregulated levels of plastid-encoded polymerase (PEP) responsible exclusively for the expression of chloroplast-encoded photosynthetic genes [65,66]. The upregulation of pTac 16 in *npp1* mutant plants under HT + ECO_2_ could at least partly explain the enhancement of photosynthesis-related genes and activity, and plastid metabolism. Hence, we consider that the high phosphorylation status in *npp1* under HT and ECO_2_ could be related to the activation of growth and carbohydrate accumulation of the seedlings.

## 3. Material and Methods

### 3.1. Plant Material and Growth Condition

The rice variety used in this study was *Oryza sativa* L. cv. Nipponbare. The *Tos17*-inserted line of *NPP1* (ND8012) was obtained from the National Institute of Agrobiological Sciences (NIAS, Tsukuba, Japan; [67]), and the *npp1-1-1* line with a single copy of *Tos17* inserted into the *NPP1* gene was established previously. WT and *npp1* mutant plants were grown and harvested at Niigata University paddy field (Niigata, Japan). 

WT and *npp1* mutant seeds were grown in a commercial soil (Kumiai Gousei Baido 3, JA, Tokyo, Japan) in plastic pots and incubated in the growth chamber (CFH-415, Tomy Seiko, Tokyo, Japan) at 28 °C (14 h day)/23 °C (10 h night) cycles with fluorescent lighting (300 μmol·m^–2^·s^–1^). Seeds and plant samples were stored at 4 °C before analysis.

### 3.2. Thermal Imaging

Thermal images of WT and *npp1* mutant plants were obtained using the InfRec (NEC) thermal video system. Plants grown for 60 days on soil (Kumiai Gousei Baido 3) were transferred to Biotron LPH-1.5PH-NCII (length 4.5 × width 4.5 × height 1.8–2.2 m, Nihon-ika, Osaka, Japan) and incubated under four different conditions (28 °C, 40 Pa CO_2_; 28 °C,160 Pa CO_2_; 33 °C, 40 Pa CO_2_; 33 °C/160 Pa CO_2_) at 70% relative humidity under natural light. 

### 3.3. Gas Exchange Measurements

Photosynthetic rate (*A_n_*), leaf conductance (*g_s_*), and intercellular CO_2_ concentrations [CO_2_] (*C_i_*) were measured with a portable photosynthesis LI-6400XL system (LI-6400-20, LiCor Biosciences, Lincoln, NE, USA). Gas exchange of WT and *npp1* leaves was recorded in the central segment of top leaves attached to the main culm between 3 and 8 h after the start of the photoperiod. Leaf cuvette conditions were set as follows: block temperature was set at ambient (growth chamber; 28 °C) and high (33 °C) temperatures; [CO_2_] was set at 400 to 2000 µmol·mol^−1^; relative humidity was maintained equal to that in the growth chamber; and Photosynthetically Active Radiation (PAR) was set at 1200 µmol·m^−2^·s^−1^, resulting in light-saturated photosynthesis and no decline as a result of photorespiration.

### 3.4. Assay

Sucrose content was measured according to the methods described previously [18,68]. Chlorophyll extracted from the isolated chloroplasts with 80% (*v*/*v*) acetone was assayed by the method described by Porra et al. [69]. Protein concentration was determined by the Pierce 660 nm Protein Assay Kit (Thermo Fisher Scientific, Waltham, MA, USA) using bovine serum albumin (BSA) as a standard.

### 3.5. Analysis of Leaf Proteome

Two hundred milligrams of leaves of WT and *npp1* grown for 7 days under normal (28 °C, 14 h day/23 °C, 10 h night, 40 Pa CO_2_) and elevated temperature and CO_2_ (33 °C, 14 h day/28 °C, 10 h night, 160 Pa CO_2_) conditions were ground in liquid nitrogen and suspended in 7 M urea, 2 M thiourea, 3% (*w*/*v*) CHAPS (3-((3-cholamidopropyl) dimethylammonio)-1-propanesulfonate), 1% (*v*/*v*) Triton X-100, and 10 mM dithiothreitol. After centrifugation at 10,000× *g* at 4 °C for 5 min, the supernatants were mixed with 1/10 volume of 100% (*w*/*v*) TCA, incubated on ice for 15 min, and then centrifuged at 10,000× *g* at 4 °C for 15 min. The resulting precipitates were washed three times with ice-cold acetone and resuspended in 8 M urea. 

The procedure of quantitative shotgun proteomic analysis was the same as previously described [68]. The protein samples (50 µg) were thoroughly digested with endoproteinase Lys-C and trypsin at 37 °C for 12 h. iTRAQ labeling of peptides was carried out with 4-plex iTRAQ tags according to the manufacturer’s protocol (AB Sciex, Framingham, MA, USA), and the resultant 4-iTRAQ-labeled peptide samples were mixed. iTRAQ analysis was performed by employing a DiNa-A-LTQ-Orbitrap-XL system operated with Xcalibur 2.0 software (Thermo Fisher Scientific). Proteins were identified with Proteome Discoverer v. 1.4 software, and the SEQUEST HT (Thermo Fisher Scientific) and MsAmanda [70] search tool using the UniProt (http://www.uniprot.org/) *O. sativa* subsp. japonica database (63,535 proteins) with the following parameters: enzyme, trypsin; maximum missed cleavages site, 2; peptide charge, 2+ or 3+; MS tolerance, 5 ppm; MS/MS tolerance, ±0.5 Da; dynamic modification, carboxymethylation (C), oxidation (H, M, W), iTRAQ 4-plex (K, Y, N-terminus). False discovery rates were <1%. 

The mass spectrometry proteomics data (JPST000338) have been deposited in the jPOST repository (https://repository.jpostdb.org/).

### 3.6. Analysis of Chloroplast Phosphoproteome

The procedure of chloroplast isolation was essentially identical to the method described earlier [16,71]. Rice seeds were germinated and grown for 7 days under normal (28 °C, 14 h day/23 °C, 10 h night, 40 Pa CO_2_) and elevated temperature and CO_2_ (33 °C, 14 h day/28 °C, 10 h night, 160 Pa CO_2_) conditions. Thirty grams of leaves were homogenized with an equal volume of solution A mixture consisting of 50 mM HEPES-KOH pH 7.5, 0.33 M sorbitol, 5 mM MgCl_2_, 5 mM MnCl_2_ and 5 mM EDTA (ethylenediaminetetraacetic acid), 50 mM sodium ascorbate, and then the homogenates were passed through four layers of gauze and four layers of Miracloth (Merck, Darmstadt, Germany). The filtrate was layered onto an 80% (*v*/*v*) Percoll (Sigma, St.Louis, USA) cushion containing solution A, and centrifuged at 2000× *g* at 4 °C for 4 min. The crude chloroplasts on the Percoll surface were diluted with more than twice the volume of solution A, then layered onto a discontinuous density gradient consisting of 40% and 80% Percoll solutions. The gradient was centrifuged at 4000× *g* at 4 °C for 10 min. Intact chloroplasts enriched around the 40%/80% Percoll interface were collected and subjected again to the Percoll gradient centrifugation. Intact chloroplasts were diluted with five times the volume of solution A, and centrifuged at 2000× *g* at 4 °C for 4 min, followed by chlorophyll and protein extractions. 

Analyses of phosphoproteins were carried out according to the method described by Fukuda et al. [72]. Intact chloroplasts were suspended in 7 M urea, 2 M thiourea, 3% (*w*/*v*) CHAPS, 1% (*v*/*v*) Triton X-100, and 10 mM dithiothreitol. After centrifugation at 10,000× *g* at 4 °C for 5 min, the supernatants were mixed with 1/10 volume of 100% (*w*/*v*) TCA, incubated on ice for 15 min, and then centrifuged at 10,000× *g* at 4 °C for 15 min. The resulting precipitates were washed three times with ice-cold acetone and resuspended in 8 M urea. The protein preparations (100 µg) were digested with 2% (*w*/*w*) endoproteinase Lys-C and trypsin in 25 mM NH_4_HCO_3_ and 0.8 M urea at 37 °C for 12 h. The reaction mixtures were dried on a Centrifugal Concentrator (CC-105, Tomy, Japan) and then dissolved in buffer A consisting of 60% (*v*/*v*) acetonitrile (ACN), 5% (*v*/*v*) glycerol, 0.1% (*v*/*v*) Trifluoroacetic acid (TFA). A MonoSpin TiO column (1000 μL: GL Science, Tokyo, Japan) was pre-equilibrated with buffer B consisting of 80% (*v*/*v*) ACN, 0.1% (*v*/*v*) TFA, followed by buffer A, and the samples were centrifuged at 3000× *g* for 1 min each. The obtained peptide samples were applied to the tip column and centrifuged at 3000× *g* for 5 min. The flow-through fraction was applied to the tip column again. Subsequently, the tip column was washed three times with buffer A and centrifuged at 3000× *g* for 1 min. The binding phosphopeptides were eluted with 100 μL of 5% NH_4_OH with centrifugation at 1000× *g* for 5 min. After elution with 5% NH_4_OH, the tightly binding phosphopeptides were eluted with 100 µL of 1 M (*w*/*v*) bis-Tris propane with centrifugation at 1000× *g* for 1 min. The phosphopeptides eluted with 5% NH_4_OH solution were dried on a Centrifugal Concentrator and then dissolved in 5% (*v*/*v*) formic acid (FA). The phosphopeptides eluted with 1 M bis-Tris propane were acidified with 900 μL of 5% FA and then desalted using a MonoSpin C18 column (GL Sciences, Tokyo, Japan).

Each phospho-peptide fraction was loaded on a HiQ sil C-18 W-3 trap column with buffer C consisting of 0.1% (*v*/*v*) FA and 2% (*v*/*v*) ACN using a DiNa-A system (KYA Tech., Tokyo, Japan). A linear gradient from 0% to 33% buffer D consisting of 0.1% FA and 80% ACN for 600 min, 33% to 100% D for 10 min and back to 0% D in 15 min was applied, and peptides eluted from the HiQ sil C-18 W-3 column were directly loaded on a MonoCap C18 High Resolution 2000 separation column. The separated peptides were introduced into a LTQ-Orbitrap XL mass spectrometer (Thermo Fisher Scientific, Waltham, MA, USA) with a flow rate of 300 nL·min^−1^ and an ionization voltage of 1.7–2.5 kV. The mass range selected for MS scan was set to 350–1600 *m*/*z* and the top five peaks were subjected to MS/MS analysis. The full MS scan was detected in the Orbitrap, and the MS/MS scans were detected in the linear ion trap. The normalized collision energy for MS/MS was set to 35 eV for collision-induced dissociation (CID).

Operation of protein identification with software and database was carried out as described above [68]. The phosphorylation of S/T/Y and oxidation of H/M/W residues were set as dynamic modifications. False discovery rates were <1%.

The chloroplast phosphoproteome data (JPST000462) have been deposited in the jPOST repository (https://repository.jpostdb.org/).

## 4. Conclusions

Previous investigations have revealed that *NPP1* exerts a negative effect on starch accumulation and growth. *NPP1* localizes to the chloroplasts and degrades a number of nucleotides including ADP-glucose and ATP, thus, it is possibly a kind of room (for example, chloroplast stroma) cleaning enzyme. The molecular physiological phenotype of the *npp1* mutant was further analyzed in the present study. Lower temperatures and changes in the transpiration rates of *npp1* leaves were observed, indicating that the disruption of the *NPP1* gene caused the stomatal opening of rice leaves. Furthermore, the analysis of the *A_n_*/*C*_i_ curve indicated that the enhancement of photosynthesis in *npp1* resulted from multiple causes in addition to the stomatal conductance. The proteome of carbohydrate metabolism and protein synthesizing system in *npp1* leaves was strongly upregulated by HT and ECO_2_. Furthermore, the protein phosphorylation status in *npp1* chloroplasts was significantly higher than in WT chloroplasts. An increase in ATP in chloroplasts might be a key stimulus, because *NPP1* preferentially hydrolyzes ATP. Judging from the overall results, we consider that the remarkable enhancement of plant growth and carbohydrate accumulation in *npp1* mutant plants under HT and ECO_2_ conditions was a consequence of the non-controlled activation of photosynthesis and protein synthesis by loss of function of a fine-tuning enzyme *NPP1*.

## Figures and Tables

**Table 1:** Phosphopeptide detection in WT and *npp1* chloroplasts with or without HT and ECO_2_ treatment. n.d.: not detected.

Description	Accession	WT Control	WT HT&ECO_2_	*npp1* Control	*npp1* HT&ECO_2_
Chlorophyll a/b-binding protein	Q6Z411	(1.15 ± 0.78) × 10^8^	(2.43 ± 2.06) × 10^8^	(1.05 ± 0.53) × 10^9^	(7.43 ± 4.27) × 10^8^
Chlorophyll a-b binding protein 2	P12331	n.d.	(4.84 ± 2.39) × 10^6^	n.d.	(4.29 ± 4.18) × 10^6^
Chlorophyll a-b binding protein	Q7XV11	(1.71 ± 1.01) × 10^6^	(3.83 ± 0.95) × 10^6^	(1.10 ± 0.44) × 10^7^	(9.44 ± 4.78) × 10^6^
Ribulose bisphosphate carboxylase large chain	P0C512	(1.08 ± 0.97) × 10^7^	n.d.	(1.52 ± 0.29) × 10^7^	(1.04 ± 0.78) × 10^7^
ATP synthase subunit beta	P12085	(3.65 ± 2.51) × 10^6^	n.d.	n.d.	n.d.
PLASTID TRANSCRIPTIONALLY ACTIVE 16	Q0DJF9	(2.55 ± 1.90) × 10^7^	(2.05 ± 1.54) × 10^7^	(0.81 ± 1.17) × 10^7^	(5.19 ± 7.87) × 10^7^
protein CURVATURE THYLAKOID 1A	Q5Z6P4	(8.76 ± 1.51) × 10^7^	(3.11 ± 0.67) × 10^7^	(2.55 ± 0.04) × 10^7^	(6.79 ± 4.23) × 10^7^
PLASTID MOVEMENT IMPAIRED1	Q0IZR7	n.d.	n.d.	n.d.	(4.16 ± 1.49) × 10^6^
Pyruvate, phosphate dikinase 1	Q6AVA8	(3.35 ± 2.78) × 10^5^	(4.62 ±0.02) × 10^5^	(1.35 ± 1.24) × 10^6^	n.d.
